# Change in Glycemic Control for Patients Enrolled in a Membership-Based Primary Care Program: Longitudinal Observational Study

**DOI:** 10.2196/27453

**Published:** 2021-06-11

**Authors:** Lenard I Lesser, Raj Behal

**Affiliations:** 1 One Medical San Francisco, CA United States

**Keywords:** diabetes mellitus, primary care, chronic care model, diabetes, self-management, patient, observational, digital health, decision support, decision-making, clinical information system

## Abstract

**Background:**

Both primary care practices based on the chronic care model (CCM) and digital therapeutics have been shown to improve the care of patients with diabetes.

**Objective:**

The aim of this observational study was to examine the change in diabetes control for patients enrolled in a membership-based primary care service that is based on the CCM.

**Methods:**

Using a diabetes registry, we analyzed the change in glycated hemoglobin (HbA_1c_) for patients with uncontrolled diabetes mellitus (initial HbA_1c_≥9%). All patients had access to a technology-enhanced primary care practice built on the CCM.

**Results:**

The registry included 621 patients diagnosed with uncontrolled diabetes. All patients had at least two HbA_1c_ measurements, with the average time between the first and last measurement of 1.2 years (SD 0.4). The average starting value of HbA_1c_ was 10.7, which decreased to 8.7, corresponding to a reduction of 2.03 (*P*<.001). Secondary analyses showed statistically significant reductions in total cholesterol, high-density lipoprotein cholesterol, low-density lipoprotein cholesterol, and triglycerides.

**Conclusions:**

Patients with initially uncontrolled diabetes who undergo care in a technology-enhanced primary care practice based on the CCM have long-term clinically meaningful reductions in HbA_1c_.

## Introduction

Uncontrolled diabetes mellitus (DM) has serious complications, including increasing the risk for heart disease, peripheral vascular disease, and kidney disease [1]. Most patients with DM are treated in primary care [2]; yet, traditional models of primary care often do not have the adequate resources to manage this chronic disease.

The chronic care model (CCM) has been described as a way for primary care practices to control chronic diseases, including diabetes [3,4]. The CCM comprises 6 components that are hypothesized to affect functional and clinical outcomes associated with disease management: (1) health system—organization of health care, (2) self-management support, (3) decision support, (4) delivery system design, (5) clinical information systems, and (6) community resources and policies.

One Medical is a membership-based (US $199/year) primary care practice based in urban and suburban locations throughout the United States. The practice mostly serves an insured population. The practice is a Patient-Centered Medical Home, as it is built on the core attributes of primary care, along with enhanced access, a quality-improvement structure, and some blended payments [5]. The aim of this observational study was to assess the change in diabetes control for patients receiving care in this nationwide practice. We hypothesized that patients engaging in our primary care model, built on the principles of the CCM, would have improved glycemic control.

## Methods

This was a retrospective observational analysis of primary care patients. As members of One Medical, these patients have access to all of the components of the CCM. The key features of the One Medical model and how they correspond to the CCM are shown in Table 1. All patients in our practice, including those with diabetes, have enhanced access to in-office and remote care, longer appointments, and self-management tools (eg, tracking of lab results and blood pressure on a mobile app). A patient with diabetes will have an evaluation and a care plan developed and documented in our problem-based electronic health record. Between primary care provider visits, the patient has constant virtual access to a team of clinicians, care navigators, and health coaches, all of whom are employed by One Medical. Care issues such as hypoglycemia or questions about medications can be addressed when they arise in this team-based care model.

**Table 1:** Chronic care model components of the One Medical model.

Chronic care model component	Feature of One Medical
Health system	Enhanced access with same-day primary care appointments and 24/7 access through messaging and on-demand video chat
Self-management	Mobile app, which provides care reminders and allows self-tracking of diabetes lab results and blood pressure
Decision support	In-house–built electronic health record that gives providers disease management “tips” and lab order sets
Delivery system design	Care navigators and virtual care providers who can manage patient care between visits with their primary care providers
Clinical information systems	Population health and quality improvement infrastructure that allows tracking of process metrics and outcomes

We used an existing quality-improvement registry of One Medical members with prediabetes and diabetes (N=7805) who had at least two glycated hemoglobin (HbA_1c_) lab measurements over a follow-up period of 180 days or longer as the source for our analysis. The registry is derived from our health system’s proprietary electronic health record, including all types of DM, with data collected from 2017 to 2020. The registry’s primary use is to improve the quality of care for patients with diabetes. From this registry, we selected a sample of patients whose first HbA_1c_ result in the practice showed their condition as uncontrolled (HbA_1c_≥9%). We performed a retrospective analysis of changes in lab-measured HbA_1c_ for this sample of patients with uncontrolled diabetes.

We report the basic demographics, BMI, as well as the Charleston Comorbidity Index (CCI) of our patients. The CCI is a chronic disease scoring system that is correlated with 10-year risk of mortality [6]. The CCI generally gives 1-6 points for each chronic disease a patient has and 1 point for every decade over age 50. The predicted 10-year survival is 96% for patients with a score of 1, 90% for those with a score of 2, 77% for those with a score of 3, and so on.

We analyzed the change in HbA_1c_ between the initial and last available value using a paired *t* test. In secondary analyses, we also evaluated changes in total cholesterol, low-density lipoprotein (LDL) cholesterol, high-density lipoprotein (HDL) cholesterol, and triglycerides. Very few patients had missing data, and therefore we chose to not remove any patients due to missing data. We used STATA Version 14 for all analyses.

## Results

The analysis included a dataset for 621 patients (8% of the registry of 7805 patients) who met our inclusion criteria as their diabetes was uncontrolled upon presentation. The average time between lab values was 1.2 years (SD 0.4). The characteristics of the patients at the time of data extraction from the registry are shown in Table 2. Patients had a median CCI of 2, indicating that the majority of patients had an additional chronic disease or were over the age of 50.

Table 3 shows the change in lab values. There was an overall decrease in the mean baseline and follow-up HbA_1c_, total cholesterol, LDL cholesterol, and triglycerides, with an average increase in HDL cholesterol.

**Table 2:** Characteristics of patients (N=621).

Characteristic	Value
Age (years), mean (SD)	51.2 (12.0)
Female, n (%)	189 (30.4)
BMI (kg/m^2^), median (IQR)^a^	31.5 (27.6-36.2)
CCI^b^ score, median (IQR)	2 (2-4)
Major depressive disorder, n (%)	126 (20.3)
Generalized anxiety disorder, n (%)	26 (4.2)

^a^BMI data were missing for 76 (12%) patients.

^b^CCI: Charleston Comorbidity Index.

**Table 3:** Changes in glycated hemoglobin (HbA_1c_) and cholesterol.

Variables	Patients, N	First value, mean (SD)	Latest value, mean (SD)	Mean difference (95% CI)	*P* value^a^
HbA_1c_ (%)	621	10.7 (1.44)	8.7 (2.16)	–2.03 (–1.85 to –2.20)	<.001
Total cholesterol (mg/dl)	599	192.9 (63.2)	172.7 (60.8)	–20.2 (–16.3 to –24.1)	<.001
LDL^b^ cholesterol (mg/dl)	542	103.4 (40.1)	90.7 (38.9)	–12.6 (–9.86 to –15.4)	<.001
HDL^c^ cholesterol (mg/dl)	596	43.3 (13.7)	44.5 (13.4)	+1.2 (0.60 to 1.8)	<.001
Triglycerides, mg/dl	597	263.1 (443.5)	206.0 (173.6)	–57.0 (–37.2 to –76.8)	<.001

^a^Based on a two-sided *t* test.

^b^LDL: low-density lipoprotein.

^c^HDL: high-density lipoprotein.

## Discussion

### Principal Results

This analysis shows that a technology-enhanced primary care practice based on the principles of the CCM can effectively control diabetes. As hypothesized, patients who had uncontrolled diabetes on initial presentation showed a marked reduction in HbA_1c_ after engaging in technology-enhanced primary care that utilizes the CCM.

### Comparison With Prior Work

Other technology-enabled diabetes interventions outside of primary care settings have also shown promise. A program focusing on diets and virtual coaching showed a 1.3% reduction in HbA_1c_ over 1 year [7]. A similar program delivered via a mobile app showed a reduction of 1.1% over 12 weeks for patients who had an initial HbA_1c_ >7% [8]. A remote monitoring intervention for diabetes with an average starting HbA_1c_ of 8.5% showed a 1% decrease in HbA_1c_ over 12 weeks [9]. The same program showed a nonsignificant decrease of 0.66% HbA_1c_ at 1 year [10]. However, patients on insulin, who started with a higher HbA_1c_, had a statistically significant 0.9% decrease in HbA_1c_.

In comparison to the above studies, our population showed a reduction of 2% in HbA_1c_. This improvement is clinically meaningful as each 1% reduction in mean HbA_1c_ is associated with relative risk reductions of 21% for deaths related to diabetes, 14% for myocardial infarction, and 37% for microvascular complications [11]. The likely reason we were able to show larger reductions was because our program combined two methods that have been shown to improve diabetes care: technology-delivered care and care based on the CCM. The above-cited studies used technology and remote care, but were not integrated in a primary care practice. Incorporating a diabetes management program with primary care practices enables accessing additional patient touch points and engagement.

A systematic review has shown that the CCM is being used across the United States to treat diabetes in primary care, with some large interventions showing improvement in diabetes control [4,12]. However, one study in the Netherlands using the CCM found reductions in cardiovascular risk factors, but not improvement in HbA_1c_ [13]. One study on chronic care clinics showed that the reduction of HbA_1c_ was dependent on the number of interactions with the patient [14]. Other research has shown that simply displaying reminders to providers in the electronic health record can improve process metrics, but not glycemic control [15,16]. Yet, increasing provider continuity—the basis of good primary care—does seem to improve disease control [17]. Further, adding specialty care to primary care does not seem to improve the control of diabetes [18]. Ease of access and the availability of a smartphone app for patient-provider communication may have contributed to effective diabetes control in our CCM-based care model. Our results provide further evidence that implementing the CCM for diabetes care in primary care can improve diabetes control.

### Limitations

This study’s design is the main limitation, as it was observational. It is possible that these patients would improve with any primary care model, even one that does not include the components of the CCM. Since we selected patients who had uncontrolled diabetes with a high baseline HbA_1c_, regression to the mean may explain some of the reductions in HbA_1c_ we observed. However, if the underlying theory of the CCM is correct, then any practice that incorporates the key features of the CCM, such as ours, should improve care.

The anticipated reduction in future cardiovascular risk may be further enhanced by lowering of LDL cholesterol as noted in this analysis. Although we did not analyze medications in this study, it is likely that many of these patients were placed on a statin. The likely reason for continued elevations in triglycerides is that our practice’s standard procedure is to test nonfasting cholesterol levels. Thus, the levels would be higher than observed in previous studies that found a correlation between triglycerides and elevated cardiovascular risk. Even if these levels confer an elevated cardiovascular risk, triglycerides have a very small independent effect on this risk [19].

Findings such as the higher variance in follow-up HbA_1c_ than in the initial measurement could not be further assessed due to the limited number of variables included in this dataset. This can be common in studies where some individuals respond to treatment and others do not. Future research should assess potential contributors to variance in similar cohorts.

### Conclusions

We demonstrated that patients with uncontrolled diabetes who receive ongoing care in a technology-based primary care model can achieve marked improvements in glycemic control. With sustained improvements, these patients will have a reduced risk for the micro- and macrovascular complications of diabetes attributed to glycemic control. This study provides further evidence that primary care practices that adopt the CCM as a model of care can improve diabetes care.

## Abbreviations

CCI: Charleston Comorbidity Index
CCM: chronic care model
DM: diabetes mellitus
HbA_1c_: glycated hemoglobin A1c
HDL: high-density lipoprotein
LDL: low-density lipoprotein
